# Cutaneous Leishmaniasis in Suspected Patients Referred To the Center for Research and Training in Skin Diseases and Leprosy, Tehran, Iran from 2008 To 2011

**Published:** 2013

**Authors:** Akram MIR AMIN MOHAMMADI, Ali KHAMESIPOUR, Alireza KHATAMI, Amir JAVADI, Mansour NASSIRI-KASHANI, Alireza FIROOZ, Yahya DOWLATI, Massoud BEHNIA, Seyyed Ebrahim ESKANDARI

**Affiliations:** 1 Center for Research and Training in Skin Diseases and Leprosy, Tehran University of Medical Sciences, Tehran, Iran; 2 Department of Community Medicine, School of Medicine, Qazvin University, Qazvin, Iran; 3 Department of Medical Parasitology and Mycology, School of Public Health, Tehran University of Medical Sciences, Iran

**Keywords:** Cutaneous leishmaniasis, Diagnosis, PCR, Leishmania species

## Abstract

**Background:**

Cutaneous leishmaniasis (CL) is a major health problem in many parts of Iran, although diagnosis of CL especially in the endemic area is easy, but treatment and management of the disease is a global dilemma. Diagnosis of CL in non-endemic area is not as simple as in endemic foci. In this study, the status and the proportions of CL induced by *Leishmania major* and *L. tropica* among CL suspected patients referred to the Center for Research and Training in Skin Diseases and Leprosy, (CRTSDL) during 2008 to 2011 are described.

**Methods:**

CL patients with suspected lesions were clinically examined. History of trip to zoonotic CL and/or anthroponotic CL endemic areas and the characteristics of their lesion(s) were recorded. Diagnosis of the lesion was done using direct smear microscopy, culture and conventional polymerase chain reaction (PCR).

**Results:**

A total of 404 (M = 256, F = 148) patients with 776 lesions were recruited and parasitologically examined. The results showed that 255 of the patients with 613 lesions; patients with lesion(s) induced by *L. major*=147 (M = 63, 43%, F = 84, 57%) and lesion(s) induced by *L. tropica*=108 (M = 35, 32%, F = 73, 68%). History of travel to endemic area was not always correlated with isolated *Leishmania* species.

**Conclusion:**

Although travel history to endemic area is an important factor to be considered for diagnosis, but parasitological confirmation is necessary initiation of treatment.

## Introduction

Leishmaniasis is a neglected tropical disease which is reported from 98 countries. Cutaneous leishmaniasis (CL) is endemic in 77 and is a major health problem in 7 countries. Ninety percent of the world CL cases are reported from Afghanistan, Algeria, Brazil, Iran, Peru, Saudi Arabia and Syria (1, 2). Leishmaniasis is a group of diseases with diverse clinical manifestations which might be categorized as cutaneous leishmaniasis, kala-azar or visceral leishmaniasis (VL), mucocutaneous leishmaniasis (MCL) and post kala-azar dermal leishmaniasis (PKDL) (3). Leishmaniasis control measures are not always effective and so far no vaccine is available against any form of leishmaniasis (4–7). VL and CL are endemic in most parts of Iran where zoonotic cutaneous leishmaniasis (ZCL) is caused by *Leishmania major* and anthroponotic cutaneous leishmaniasis (ACL) is caused by *L. tropica*, CL is endemic in 17 of 31 provinces and is a major health problem of the country (8).

Treatment of CL is not easy especially when the causative agent is *L. tropica*. Although pentavalent antimonite derivatives are still considered as the main treatment available for CL, their efficacies are not promising and resistance has been reported. Maglumine antimoniate (MA) (Glucantime^®^ Rhodia Laboratories, Rhone-Poulenc, France) is considered as the only standard treatment available for CL in Iran. However, MA treatment is administered via parenteral route which needs multiple painful injections, is accompanied by several side effects and questionable efficacy (9–13). Diagnosis of CL in its typical form in endemic area is not difficult, but atypical forms of the disease resemble various skin disorders such as furuncle, ecthyma, tuberculosis, atypical mycobacterial infections, deep mycosis, sarcoidosis, leprosy, syphilis, foreign body granuloma and even sometimes malignant skin tumors which justify to initiate the treatment only when the lesion is parasitologically confirmed. Diagnosis of CL is based on the presence of *Leishmania* amastigotes in a direct smear prepared by scratching of the periphery of a suspected lesion (14). Usually, research facilities in endemic areas perform culture on Novy–Nicolle–McNeal (NNN) medium in addition to direct smear. Molecular techniques such as polymerase chain reaction (PCR) are also used for diagnosis of CL and identification of the species, but need infrastructures which do not exist in most endemic areas. Center for Research and Training in Skin Diseases and Leprosy (CRTSDL) is the first skin diseases research center in Iran which established as a referral center for skin diseases, including diagnosis and treatment of CL. In Cutaneous Leishmaniasis Clinic of the CRTSDL diagnosis and treatment and follow up of the patients are done free of charge for all the patients. PCR for diagnosis of *Leishmania* infection is rarely done but in the Cutaneous Leishmaniasis Clinic of the CRTSDL, PCR is routinely done for diagnosis and identification of *Leishmania* due to the fact that as a reference center numerous patients are referred by dermatologists from all over the region.

The aim of this study was to describe the CL patients and to determine proportion of ACL and ZCL cases among the CL suspected patients who were referred to the CRTSDL from 2008 to 2011.

## Materials and Methods

### Sample collection

The procedure was based on National (Iranian) Protocol for Diagnosis and treatment of Cutaneous Leishmaniasis which has been developed in accordance with World Health Organization (WHO) recommendations. Every patient was interviewed and his/her lesion (s) characteristics were recorded and photographed. One lesion from each patient, usually the largest one, was selected and the lesion and its surrounding skin were cleaned and sterilized using 70° ethylic alcohol. Samples were collected by scrapping of the skin from the margins of the lesion and three samples were collected and used for direct smear, culture and PCR.

### Direct Smear

The sample was smeared on a glass slide and stained using Giemsa (10%) stain, and then the slide was checked under microscope in search for *Leishmania* amastigotes, sometimes sampling was done more than once, twice or thrice when the first attempt failed to see the parasite, slide was not taken appropriately or when the sample showed super infection.

### Culture

A sample from each patient was transferred into NNN medium and incubated at 26 ± 1 °C and the growth of promastigote was checked every 4 days for 2 weeks, when promastigote growth was seen, the sample was subcultured in RPMI 1640 medium (Gibco, UK) supplemented with 10% heat-inactivated fetal calf serum (Gibco, UK), streptomycin (20 mg/ml) and penicillin (100 IU/ml), and incubated at 26 ± 1 °C.

### DNA extraction

DNA extraction was done, the suspected sample was added to 200 µl of lysis buffer (100 mM Tris; 1% SDS; 10 mM EDTA; 100 mM NaCl) and 20 µl Proteinase K and incubated at 56 °C for 60 min, 300 µl phenol-chloroform (50:50 v/v) was added to lysate's micro tube and centrifuged for 5 min at 5,000 rpm in a micro centrifuge. The upper layer was added to an equal volume of phenol and centrifuged for 5 min at 5,000 rpm. The supernatant was added to an equal volume of isopropanol and 1/10 volume of sodium acetate. Following incubation at -20°C for 10 min, the sample was centrifuged at 12,000 rpm for 15 min. The pellet was washed in 300 µl 70% ethanol and centrifuged at 5,000 rpm for 5 min and then the pellet was resuspended in 20 µl of sterile distilled water (DW) and stored at -20 °C until use.

### PCR amplification

One µl of each extracted DNAs and 1 µl a pair of primers was added to 12.5 µl Master Mix and distilled water in 25 µl volume and microtubes were then placed in Eppendorf Mastercycler Gradient set for DNA amplifying. The sequences of the primers used are as follow 5’ TCGCAGAACGCCCCTACC 3’ and 5’ AGGGGTTGGTGTAAAATAGG 3’ (Cinnagene, Iran) (15).

A first denaturation step of 5 minutes at 95°C was followed by amplification for 35 cycles: 30 second at 94°C for denaturation, 45 second at 60°C for annealiation and 1 minute at 72°C for elongation using DNA polymerase and finally a single 5 minutes cycle at 72°C for final elongation. The PCR products were examined using electrophoresis on 1.5% agarose gel, agarose gel stained with ethidium bromide, using a 100 bp DNA ladder as a marker and visualized using a UV transluminator. Every PCR reaction included 3 positive (*L. major*, *L. tropica* and *L. infantum* species) and a negative control.

### Statistical analysis

Version 12 of SPSS (SPSS Inc., Chicago, IL, USA) software was used to analyze the data. Data were summarized using mean + standard deviation (SD) for those had a Normal distribution and median (interquartile range) for nonparametrical ones. The summarized data were provided in tables. Kruskal-Wallis test was used to detect significant differences in means. A *P* value of less than 0.01 was considered to be significant.

## Results

A total of 404 [F = 148, (36.5%), M = 256, 63.5%)] patients with 776 suspected CL lesions from different parts of Iran and neighboring countries were included. The patients were from all age ranges (1-93 years old, median = 29 years, mean ± SD = 32.9 ± 18.5 years). Two hundred and twenty seven of the patients were referred to CRTSDL by dermatologists and 177 of the patients were visited in CRTSDL for the first time. The onset of the lesion was from 1 week to 21 years. Based on the histories of the patients’ trips to endemic areas, sand fly bites and exposure to *Leishmania* was speculated. Probable exposure to *Leishmania* in 247 of the patients were defined to be in endemic areas of 9 provinces of ZCL, ACL endemic areas or mixed ZCL/ACL and the causative species was identified. Four patients were from Pakistan and 30 patients from Afghanistan which with respect to their travel history most probably were exposed to sand fly bites in their own countries. No history of trip to a known endemic area was defined in 45 of the patients. Parasitological examinations including direct smear microscopy, culture and PCR were performed for all suspected CL cases and PCR confirmed CL in 255 of the patients with 613 lesions. Identification of *Leishmania* species in 255 CL cases was done. On rare occasions, mouse model was also used. The results confirmed *L. major* infection in 147 of the patients with 373 lesions (median) (inter quartalile range): [2] and *L. tropica* infection in 108 patients (median) (inter quartalile range): [1][2] and 150 patients with 164 (median) (interquartalile range): [1][1] of the patients’ lesions were not CL (Tables 1 and 2), 45 of the patients with no history of travel to endemic areas, the PCR results showed that 30 of the patients were infected with *L. major* and 15 were infected with *L. tropica*. Nine patients were residents of Tehran with no history of known trip to endemic areas, the causative agent in 7 of them was *L. tropica* and 2 were infected with *L. major*. In direct smear evaluations, amastigotes were seen in 197 of the samples and 213 of the samples cultured in NNN showed promastigote growth. All of the patients received standard treatment regimens in accordance with National (Iranian) Protocol for Diagnosis and Treatment of Cutaneous Leishmaniasis, free of charge.

**Fig. 1:**
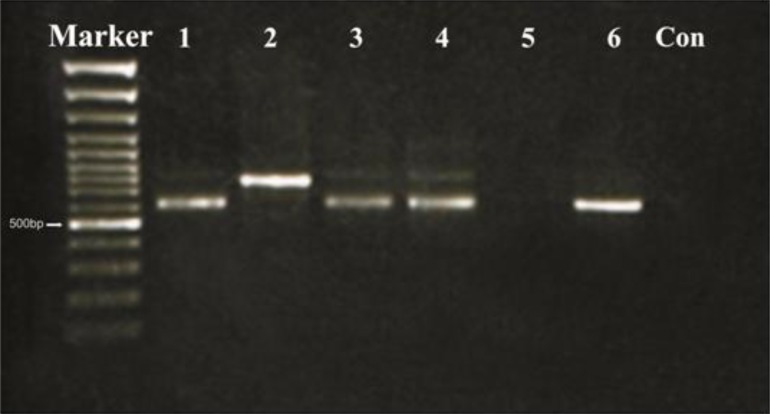
PCR results. Left to Right: Marker, 1-Pateint sample (*L.major*) 2 Positive –(*L.tropica)* 3-patient sample (*L.major*) 4- Patient sample (*L.major*) 5- Patient sample (not cutaneous leishmanisis) 6- Patient sample (*L.major*) 7-Negative Control (*L. major* 600bp and *L. tropica* 800bp)

**Table 1:** Distribution of the patients with CL caused by *L. major* or *L. tropica* and patients with lesion other than CL

Kind	Patients infected with *L.major* (%)	Patients infected with *L. tropica* (%)	Patients none CL Lesion (%)	Total
ZCL	98 (71)	3 (22)	37 (27)	138
ACL	12 (14)	48 (57)	24 (29)	84
ACL-ZCL	7 (12)	42 (71)	10 (17)	59
Unknown	30 (24)	15 (12)	78 (64)	123
Total	147	108	149	404

**Table 2:** Number of lesions in patients with cutaneous lesion(s)

No. of lesion	CL lesion and *Leishmania* species		No CL lesions
	*L. major*	*L. tropica*	
**1**	59	57	135
**2**	35	22	14
**3**	25	9	0
**4**	7	8	0
**5**	7	5	0
**6**	4	3	0
**7**	2	0	0
**8**	2	2	0
**9**	1	1	0
**10**	2	0	0
**11**	1	0	0
**12**	1	1	0
**Total**	373	240	163

## Discussion

According to WHO/EMRO leishmaniasis is reported from 18 of 23 countries of EMRO region and most of the countries neighboring Iran are endemic to leishmaniasis. CL is endemic in most parts of Iran and is a major health problem. Management of CL is also a major concern in endemic areas (16). Treatment of CL required multiple injections which are painful and are associated with potentially serious side effects, so parasitological confirmation is necessary before initiation of treatment.

Moreover, identification of the causative *Leishmania* agent of CL is an important issue since choosing treatment strategy and prognosis of the disease depend on the causative species (9, 13). Characteristics of the lesion and epidemiological information are not enough to define *Leishmania* species especially in endemic areas with mixed ACL and ZCL infections (17–21). It is recommended that treatment be initiated only when the lesion has been confirmed parasitologically. If continuation of the treatment, for example in the lesions which do not heal in an expected time period is needed, then identification of the causative agent (*L. major* or *L. tropica*, etc.) using molecular methods is required (15).

In several CL patients who were referred to the CRTSDL Cutaneous Leishmaniasis Clinic with positive smear reports, when parasitological tests including direct smear, culture and PCR were repeated, the results showed that in 4 patients the lesions were not CL, although all 4 patients presented to the clinic with positive direct smear results, This finding emphasizes that sampling and microscopic examinations for direct smear should be done by well-trained laboratory technicians. In a study performed in Mashhad (20), the sensitivity of positive direct smear result was 81% and the sensitivity of a positive culture was 84%. In the current study, the sensitivity of a positive direct smear was 77.2% and the sensitivity of a positive culture was 83.5% which was higher in comparison with a positive direct smear. It might be because of different types of patients. In the current study, culture media were kept for a long time with regular check. Median (interquartile range) of the number of lesions in ZCL was 2 and for ACL was 1 and in patients with lesions other than CL was 1. The median number of lesion was significantly (different between the groups, which indicated that if the patient suffers multiple lesions the chance that the lesions are caused by CL increases and the number of lesions in ZCL are higher than their number in ACL patients (*P*<0.001).

For the patients with a disease onset less than 2 years, Glucantime^®^ (Rhodia Laboratories, Rhone-Poulenc, France) was administered according to the National (Iranian) Guideline for Diagnosis and Treatment of Cutaneous Leishmaniasis, but the patients whose lesions’ onset was more than two years, whom usually had a history of treatment failure, were treated with Glucantime^®^ (Rhodia Laboratories, Rhone-Poulenc, France) 20 mg/kg of Sn^5 +^ up to a maximum of 3 5 ml vials per day for 14 to 21 days in combination of allopurinol 10-15 mg/kg for 4 weeks.

## Conclusion

History of travel to known endemic area or living in endemic area is important factor to suspect a lesion as cutaneous leishmaniasis, but parasitological confirmation is the only proof to initiate the treatment.
